# Immunohistochemical Detection of TAS2R38 Protein in Human Taste Cells

**DOI:** 10.1371/journal.pone.0040304

**Published:** 2012-07-06

**Authors:** Maik Behrens, Stephan Born, Ulrike Redel, Nadine Voigt, Vanessa Schuh, Jan-Dirk Raguse, Wolfgang Meyerhof

**Affiliations:** 1 Department of Molecular Genetics, German Institute of Human Nutrition Potsdam-Rehbruecke, Arthur-Scheunert-Allee 114-116, Nuthetal, Germany; 2 Clinic and Polyclinic for Oral and Maxillofacial Surgery and Plastic Surgery, Charité, Campus Virchow Hospital, Berlin, Germany; Barnard College, Columbia University, United States of America

## Abstract

The sense of taste plays an important role in the evaluation of the nutrient composition of consumed food. Bitter taste in particular is believed to serve a warning function against the ingestion of poisonous substances. In the past years enormous progress was made in the characterization of bitter taste receptors, including their gene expression patterns, pharmacological features and presumed physiological roles in gustatory as well as in non-gustatory tissues. However, due to a lack in TAS2R-specifc antibodies the localization of receptor proteins within gustatory tissues has never been analyzed. In the present study we have screened a panel of commercially available antisera raised against human bitter taste receptors by immunocytochemical experiments. One of these antisera was found to be highly specific for the human bitter taste receptor TAS2R38. We further demonstrate that this antibody is able to detect heterologously expressed TAS2R38 protein on Western blots. The antiserum is, however, not able to interfere significantly with TAS2R38 function in cell based calcium imaging analyses. Most importantly, we were able to demonstrate the presence of TAS2R38 protein in human gustatory papillae. Using double immunofluorescence we show that TAS2R38-positive cells form a subpopulation of PLCbeta2 expressing cells. On a subcellular level the localization of this bitter taste receptor is neither restricted to the cell surface nor particularly enriched at the level of the microvilli protruding into the pore region of the taste buds, but rather evenly distributed over the entire cell body.

## Introduction

The ability to taste potentially harmful bitter compounds occurring frequently in nature is important for a wide range of animal phyla ranging from insects to mammals [1]. In humans, the detection of bitter compounds is facilitated by ∼25 bitter taste receptors belonging to the TAS2R gene family of G protein-coupled receptors [2]. All TAS2Rs share a conserved site for Asn-linked glycosylation within the center of the second extracellular loop. The co-translational addition of oligosaccharide moieties at this position is indispensable for efficient functional expression of TAS2Rs [3]. An intriguing question after the initial discovery of bitter taste receptor genes [4]–[6] was, how relatively few receptors may suffice to detect countless structurally diverse bitter compounds present in nature. In particular, for humans, the successful deorphanization of 20 of the 25 TAS2Rs [2], uncovered that the repertoire of human TAS2Rs consists of some extremely broadly tuned “generalist” receptors, such as TAS2R10 [2], [7], TAS2R14 [8] and TAS2R46 [9] underlying the detection of a large fraction of all bitter compounds [2], several intermediately tuned receptors, as well as narrowly tuned “specialist” receptors, such as TAS2R3 [2] and TAS2R50 [10]. Additionally, two receptors, TAS2R16 [7] and TAS2R38 [11], are specifically tuned to detect distinct classes of bitter chemicals, such as β-D-glucopyranosides and isothiocyanates, respectively. It has been observed that TAS2Rs form homo- and heteromers if co-expressed *in vitro*. However, at present, no evidence for functional implications arising from receptor oligomerization exists [12].

On a cellular level, bitter taste receptor genes are expressed within taste buds in a distinct subpopulation of type II taste receptor cells, not overlapping with those type II taste receptor cells devoted to the detection of sweet and umami stimuli (for a recent review see [13]). Each bitter taste receptor cell in human circumvallate papillae expresses several, but not all, TAS2R genes, accounting for a heterogeneous cell population [14].

In addition to type II taste receptor cells, taste buds contain also type III cells (also known as presynaptic cells), the only cell type within taste buds forming synapses with afferent nerve fibers, the glia-like type I cells as well as stem cell-like basal cells. Each taste bud possesses a single apical pore where the microvilli of the taste cells come in contact with tastants present in the oral cavity (for a recent review see [13]). It appears conceivable that the bitter taste receptor proteins should be present within the microvillar membranes to facilitate the detection of tastants, although alternative scenarios where tastants rapidly diffuse into the taste receptor cells, e.g. leading to the direct activation of second messenger cascades are discussed for amphipathic molecules [15]. However, due to a lack of specific antibodies against bitter taste receptor proteins, expression studies relied until now on the detection of mRNA by *in situ* hybridizations [4], [6], [14]. Thus, although discovered more than a decade ago, the distribution of bitter taste receptors within their natural cellular environment is still somewhat speculative.

In recent years, numerous antibodies raised against bitter taste receptor proteins of various species became commercially available. As we are particularly interested in human bitter taste perception, we evaluated several antibodies raised against human TAS2Rs for their suitability for expression studies in human gustatory tissues. We tested the ability of the antibodies to detect human TAS2Rs first by immunocytochemical experiments using transiently transfected mammalian cells. The single antibody that exhibited reliable specific detection of the transfected receptor protein was further used for immunofluorescent staining of human circumvallate papillae sections, Western blotting and functional calcium imaging experiments.

## Results

**Figure 1:**
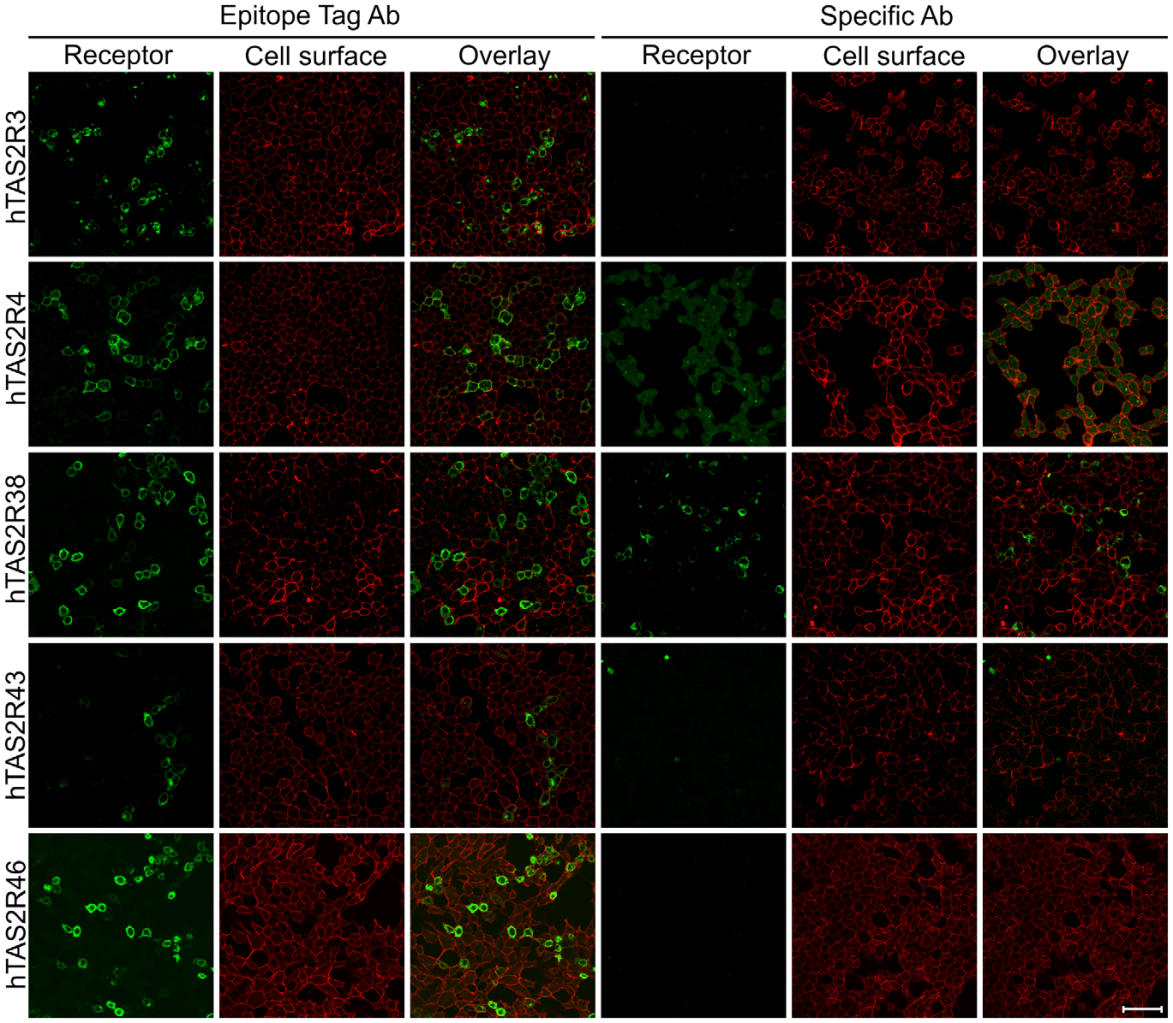
Screening for human bitter taste receptor-specific antibodies by immunocytochemistry HEK 293T-Gα16gust44 cells were transiently transfected with cDNA coding for the human bitter taste receptors TAS2R3, -R4, -R38, -R43, and -R46. To visualize the receptor proteins (Receptor, green) either an antibody against the C-terminal HSV-tag (left panels, Epitope Tag Ab) or commercially available specific antibodies were used (right panels, Specific Ab). The cell surfaces were labeled with biotin-conjugated concanavalin A (Cell surface, red). Overlay pictures of the green and red channels are shown to visualize the amount of receptor at the level of the plasma membrane (Overlay). Scale bar, 50 µm.

For an initial screening of the versatility of antisera raised against TAS2R proteins we used HEK 293T-Gα16gust44 cells transiently transfected with cDNA of TAS2R3, -R4, -R38, -R43, and –R46. The transfected cells were then stained using antibodies reported to specifically detect human bitter taste receptors TAS2R4, -R38, -R43, and –R46 in airway epithelia [16]. In addition, an antiserum raised against human TAS2R3 was included in this screening. In order to allow an independent monitoring of transfection rates/expression frequencies on identically treated cells we used in addition an antibody specific for the *Herpes simplex* virus glycoprotein D-epitope (HSV-tag, TAS2R3, -R4, -R38, -R43) or an antibody recognizing a FLAG-tag (TAS2R46). Both epitope-tags/antisera have successfully been used for the immunocytochemical detection of heterologously expressed bitter taste receptors in the past [7], [8], [10], [17]. As shown in figure 1, all TAS2R proteins are detected if using the anti-HSV/anti-FLAG antisera (Fig. 1, left panels). Comparing the number of cells stained for the receptor proteins (green) with the staining of cell surfaces using concanavalin A (red) demonstrated that for all transfected receptors except TAS2R43 a comparable fraction of cells were labeled. Even though the apparent expression rate of TAS2R43 in this experiment was reduced compared to the other four receptors, numerous green cells confirm that the transfection and detection was successful. In contrast to the immunocytochemical detection via the C-terminal epitopes, the TAS2R antibodies worked less consistently (Fig. 1, right panels). Whereas no signals were obtained using antibodies raised against TAS2R3, -R43, and –R46, the TAS2R4 antiserum exhibited an elevated background signal evident in all cells as visible from the corresponding cell surface staining. Solely the TAS2R38 antibody led to a labeling of a subset of cells similar to the staining pattern observed using the anti-HSV-antibody.

**Figure 2:**
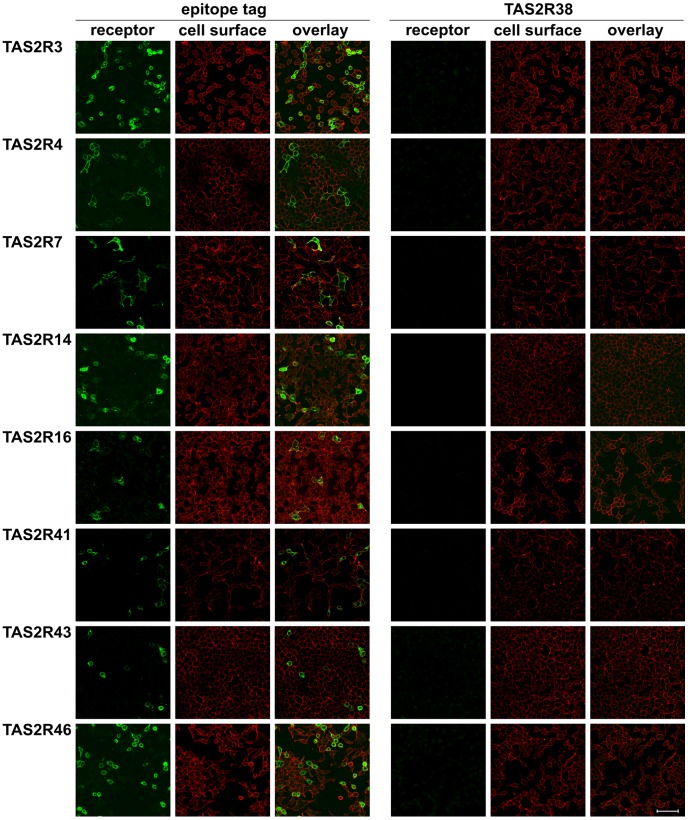
Screening for cross-reactivity of the TAS2R38 antiserum with other TAS2Rs HEK 293T-Gα16gust44 cells were transiently transfected with cDNA coding for the human bitter taste receptors TAS2R3, -R4, -R7, -R14, -R16, -R41, -R43, and -R46. To visualize the receptor proteins (receptor, green) either antibodies against the C-terminal epitope-tags (left panels, epitope tag Ab (anti-HSV antiserum for TAS2R3, -R4, -R7, -R14, -R16, -R41, -R43; anti-FLAG antiserum for TAS2R46) or the TAS2R38-specific antiserum (right panels, TAS2R38) were used. The cell surfaces were labeled with biotin-conjugated concanavalin A (cell surface, red). Overlay pictures of the merged green and red channels are shown (overlay). Scale bar, 50 µm.

As our initial screening results indicated that the TAS2R38 antibody specifically labeled cells transfected with TAS2R38 cDNA, we next investigated if the antiserum is indeed specific for this receptor or if cross-reactivity with other TAS2Rs is observed (Fig. 2). Therefore, we transfected cells with constructs coding for TAS2R3, -R4, -R7, -R14, -R16, -R41, -R43, and –R46, representing about one-third of the functional human bitter taste receptor repertoire, and performed immunocytochemical experiments as described above. Instead of using the individual antisera raised against TAS2Rs, we only used the TAS2R38 antiserum in addition to the epitope-tag selective antisera. Whereas all receptor constructs were detectable via their epitope-tags (Fig. 2, left panels) confirming their successful expression, no signals were obtained using the specific TAS2R38 antiserum (Fig. 2, right panels). Therefore, there is no indication of cross-reactivity with a large panel of TAS2Rs distinct from TAS2R38 (see Figures. S1 and S2 for results obtained with additional 10 TAS2Rs).

**Figure 3:**
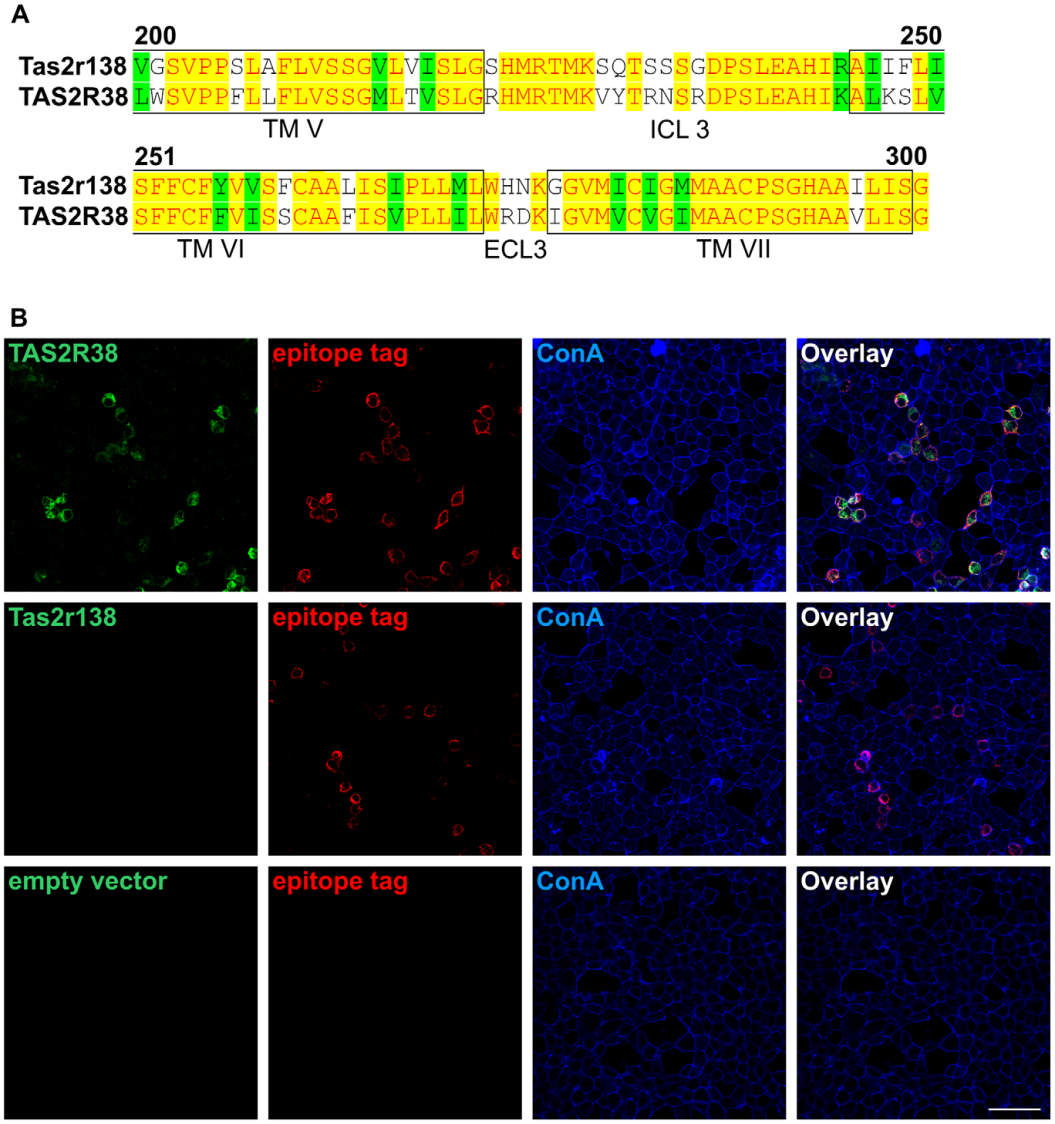
Comparison of the immunocytochemical co-staining patterns of anti-TAS2R38 and epitope tag-specific antibodies A, Comparison of the amino acid sequences of the orthologous bitter taste receptors mouse Tas2r138 (first row) and human TAS2R38 (second row). Shown are the receptor regions, which were indicated by the supplier of the TAS2R38-antiserum to contain the epitope used for antiserum production (between amino acid positions 200 and 300, extracellular). Identical amino acid residues are highlighted in yellow, similar residues in green and divergent residues in white. Transmembrane domains are boxed and labeled (TM). Intracellular loop 3 (ICL3) and the single extracellular loop contained in the region, extracellular loop 3 (ECL3), are indicated. The alignment was done with the program AlignX, a component of the Vector NTI Suite 9.0.0, and the receptor subdomains were assigned according to Biarnes et al. [40]. B, HEK 293T-Gα16gust44 cells were transiently transfected with cDNAs coding for the TAS2R38-taster variant (PAV) (top panel), the orthologous mouse receptor Tas2r138 (middle panel) as specificity control or empty vector (bottom panel) as negative control. Cells were subsequently stained in parallel with a TAS2R38-specific antibody (green), an anti-HSV antibody, (epitope tag, red) and with biotinylated concanavalin A (ConA, blue). An overlay of the green, red and blue channels is shown (Overlay). Note the almost complete overlap of green and red signals indicating that both, the epitope-specific and the TAS2R38-selective antibody, recognize an identical population of TAS2R38 transfected cells. The absence of green signals in cells transfected with mouse Tas2r138 confirms the selectivity of the anti-TAS2R38 antiserum. Cells transfected with empty expression vector exhibited neither TAS2R38- nor HSV-immunoreactivity.

Next, we wanted to find out whether the staining efficiency provided by the specific anti-TAS2R38 antiserum is comparable to the HSV-antibody we used frequently in the past. To this end, we transfected cells with TAS2R38 cDNA and performed co-immunostaining experiments. We included in this experiment the orthologous mouse bitter taste receptor Tas2r138 ( [18], former gene symbol mT2R31) that shares an overall amino acid sequence identity of 65% and is within the region used to raise the antibody (extracellular, between amino acids 200–300 of TAS2R38, cf. discussion section) even more similar (Fig. 3A). While the successful detection of both receptors, TAS2R38 and Tas2r138 would indicate that this antibody might be useful also in rodent studies, the absence of staining would indicate its pronounced specificity. The epitope tags were detected using the mouse monoclonal anti-HSV antibody (Fig. 3B, red). In parallel, the TAS2R38 and Tas2r138 proteins were visualized by the rabbit anti-TAS2R38 antiserum (Fig. 3B, green), and the cell surface was labeled by concanavalin A (Fig. 3B, blue). The overlay image of green, red and blue channels (Fig. 3B) demonstrates that virtually all cells stained with the anti-HSV antiserum are also labeled by the specific TAS2R38 antibody. Although, on a cellular level, the staining pattern of both antibodies largely overlaps, on a subcellular level the areas of predominant green or red signals indicate some minor differences in detection preferences. The complete absence of signals in cells expressing mouse Tas2r138 demonstrates the selectivity of the antiserum, however, at the same time proves that this antiserum is not a useful tool for studying bitter taste receptor expression in rodents.

**Figure 4:**
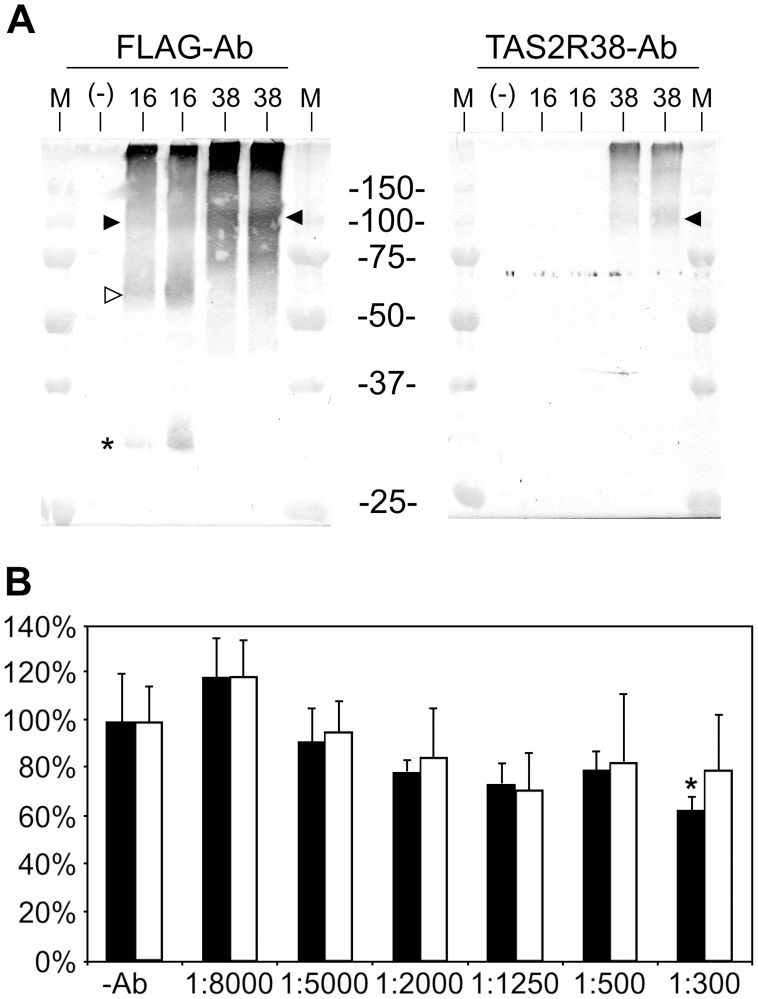
Western blotting of extracts of TAS2R38 transfected cells and functional calcium imaging analyses A, Western blotting of membrane protein extracts. HEK 293T-Gα16gust44 cells were transiently transfected with DNA for TAS2R38 C-terminally extended with a FLAG-tag (38) and subsequently used for immunoprecipitation with anti-FLAG agarose beads. As controls, FLAG-tagged TAS2R16 (16) and membrane protein extracts obtained after transfection with empty vector (-) were used in parallel. For TAS2R16- and TAS2R38-preparations the experiment was performed in duplicates. Left panel, Western blot of cell extracts visualized with an anti-FLAG antibody; right panel, Western blot of cell extracts visualized by staining with anti-TAS2R38 antibody. Open arrow heads indicate bands corresponding to the predicted molecular weight of full length glycosylated receptor monomers. Filled arrow heads point to bands corresponding to the predicted molecular weight of receptor oligomers, asterisks indicate truncated receptor variants. M =  Molecular weight standard (the sizes of the marker proteins in kDa are indicated). B, functional calcium imaging analysis of HEK 293T-Gα16gust44 cells transiently transfected with cDNA coding for TAS2R38 PAV, taster variant) and TAS2R10. Prior to stimulation with bitter compounds (PTC at a concentration of 30 µM for TAS2R38 and denatonium benzoate at a concentration of 100 µM for TAS2R10) cells were incubated with different dilutions of the TAS2R38 antibody for 30 min. The signals obtained for stimulated cells not treated with antibodies (−Ab) were set as 100%. The signals monitored after stimulations of antibody treated cells were compared to the −Ab controls. The responses of TAS2R38 (black bars) and TAS2R10 (white bars) were tested for statistical significance compared to the untreated controls using student’s t-test. The asterisk indicates a statistical significant reduction of the TAS2R38 response of cells treated with a 1∶300 dilution of the TAS2R38 antibody. However, compared to the correspondingly treated TAS2R10-control no statistically significant reduction was observed.

We next examined if the anti-TAS2R38-antiserum is able to recognize the denatured receptor protein by Western blotting. For the Western blotting we transiently transfected HEK 293T-Gα16gust44 cells with cDNA coding for TAS2R38, TAS2R16 or empty vector. In order to facilitate efficient immunoprecipitation of the receptor proteins, we used C-terminally FLAG-tagged constructs and incubated membrane protein extracts with FLAG-agarose beads. After SDS-PAGE, the anti-FLAG antibody detected immunoprecipitated receptor proteins in the two lanes loaded with the samples from TAS2R16 cell extracts as well as in the duplicate samples corresponding to TAS2R38 cell extracts (Fig. 4A, left). On the contrary, the specific anti-TAS2R38-antiserum only recognized receptor protein in samples originating from TAS2R38-transfected cells (Fig. 4A, right).

The deduced molecular weight for the N− and C-terminally tagged TAS2R16 protein is 39.6 kDa. As the sst3-tag contains two sites for Asn-linked glycosylation [19] and the TAS2R16 protein itself is glycosylated once within the 2^nd^ extracellular loop [3], the band migrating just above 50 kDa on the Western blot treated with the anti-FLAG antibody (Fig. 4A, left, open arrow head) likely corresponds to the glycosylated full length receptor monomer. Additionally, in agreement with previous observations, a slower migrating putative receptor dimer [12] (filled arrow head) as well as a faster migrating possibly truncated receptor variant [3], [17] (asterisk) is evident. The TAS2R38 protein, which has a larger deduced molecular weight (43.6 kDa including the fused epitope tags), migrates predominantly at sizes indicative of dimeric (filled arrow head) and even higher order oligomeric forms. On the Western blot treated with the specific anti-TAS2R38-antiserum (Fig. 4B, right), specific staining is restricted to lanes corresponding to TAS2R38-transfected cell extracts. In agreement with the results of the Western blot treated with the FLAG-specific antiserum, bands presumably corresponding to TAS2R38 dimers are evident.

In order to test whether the anti-TAS2R38 antiserum would interfere with the function of the receptor in calcium imaging experiments, as shown by Deshpande and colleagues for an anti-TAS2R10 antiserum blocking strychnine induced calcium responses in airway smooth muscle cells [20], we performed calcium-imaging experiments using cDNA coding for the TAS2R38-taster variant (PAV) [11], [21] and, for specificity control, the TAS2R10 (Fig. 4B). The relative increases in fluorescence upon stimulation with signal saturating concentrations of 30 µM phenylthiocarbamide (PTC) and 100 µM denatonium benzoate for the TAS2R38 and TAS2R10 [2], [11], respectively, were set as 100% activation. Stimulations after preincubation for 30 min with different dilutions of the anti-TAS2R38 antiserum were compared to the responses of identically stimulated cells not treated with antibodies. As evident from the graph shown in figure 4B, higher antibody concentrations during the preincubation tended to result in a modest and insignificant decrease of signal amplitude obtained for cells expressing TAS2R38 as well as for cells expressing TAS2R10. At a dilution of 1∶300 the anti-TAS2R38 antiserum significantly decreased the signal amplitudes of TAS2R38-expressing cells stimulated with PTC slightly, whereas TAS2R10-transfected cells did not significantly respond to antibody treatments even at the highest concentration. However, although the decrease in the signal of TAS2R38-transfected cells preincubated with the 1∶300 diluted antiserum was significant compared to cells not treated with antisera, comparison with the similarly treated TAS2R10-expressing cells did not reach significance. Thus, the TAS2R38 antibody appears to be of rather limited use as a pharmacological reagent.

**Figure 5:**
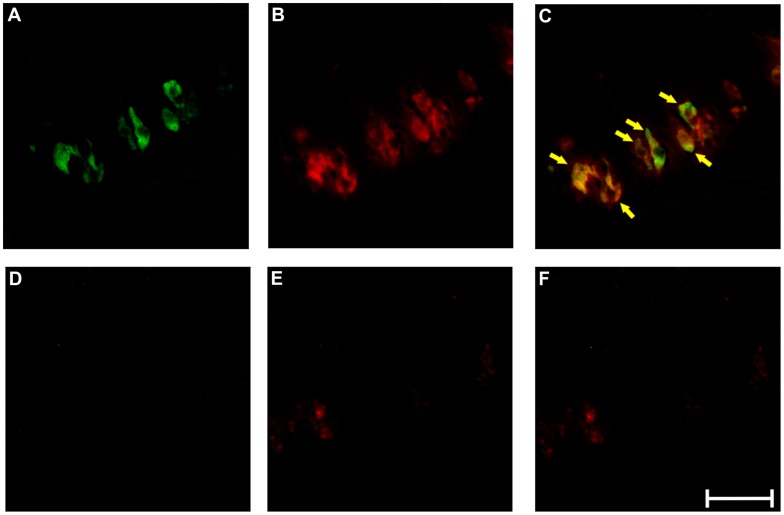
Immunofluorescent detection of TAS2R38 protein in human circumvallate papillae Cross sections of human circumvallate papillae were analyzed for the localization of TAS2R38 in type II taste receptor cells using a PLCβ2-specific antibody as cell-type marker (A-F). The bitter receptor TAS2R38 is labeled in green (A), the type II cell-specific signal transduction molecule PLCβ2 is visualized in red (B). An overlay picture of green and red channels is shown in C. Arrows point to cells co-labeled with both antisera. For specificity controls pre-absorbed antibodies using immunogenic peptides specific for anti-TAS2R38 (D) and anti-PLC β2 (E) were used on adjacent sections. An overlay picture of green and red channels is shown in F. Scale bar, 50 µm.

Having characterized the selectivity of the anti-TAS2R38 antiserum we performed immunohistochemical experiments to locate the receptor *in vivo* in human taste tissue (Fig. 5), a site known to contain TAS2R38 RNA [11], [14]. In order to exclude the possibility that antigens other than or in addition to TAS2R38 are stained in mammalian taste tissue, we tested several secondary antibody/anti-TAS2R38 combinations first on mouse vallate papillae sections, which should not show TAS2R38-immunoreactivity (cf. Fig. 3). From these results (Fig. S3) the best working antiserum combination was chosen to perform the experiment on human circumvallate papillae sections. We performed double-immunofluorescence labeling experiments using the anti-TAS2R38 antiserum in combination with a PLCβ2 antiserum to demonstrate expression of TAS2R38 in type II taste receptor cells [22]–[24]. As seen in figure 5, both antisera label subsets of cells within taste buds of human circumvallate papillae. As anticipated, signals obtained for TAS2R38 (A, green) frequently colocalize with PLCβ2-specific signals (B, red and C, overlay of A and B). Several cells that express PLCβ2 do not co-express TAS2R38 indicating that they may represent bitter taste receptor cells expressing other TAS2R-genes [14] or that they belong to the sweet [25]–[27] or umami-receptor [28], [29] expressing subsets of type II taste receptor cells. The localization of TAS2R38 is neither restricted to the extracellular surface nor the pore region of bitter taste receptor cells. The specificity of the staining procedure is demonstrated by parallel experiments in which the primary antisera have been pre-absorbed with the corresponding antigenic peptides. As expected for a specific reagent, this antiserum preparation with reduced levels of reactive specific anti-TAS2R38 antibodies did not stain cellular structures in the lingual epithelium (Fig. 5D–F).

## Discussion

Since many years the field of bitter taste research has been waiting for tools allowing the *in vivo* detection of TAS2R in mammalian gustatory tissues. Recently, numerous antisera became commercially available to close this gap. Although several of these antibodies were used to investigate bitter receptors outside the gustatory system [16], [20], [30], so far, the most obvious application of such antisera, the investigation of the gustatory expression of bitter taste receptors has not been addressed. In the present manuscript, we determined the specificity of antisera raised against five different human bitter taste receptor proteins by immunocytochemical experiments. Whereas the anti-TAS2R38 antiserum detected the corresponding receptor in transiently transfected mammalian cell lines, the other antisera failed to recognize their specific epitope even though comparatively high expression levels of the receptors likely occur under the chosen experimental conditions. In parallel experiments with antibodies recognizing C-terminal epitopes added to the receptor proteins, we were able to confirm comparable expression levels for all receptor constructs. Of course, numerous experimental circumstances can account for a failing antiserum-immunogen reaction: The epitope chosen for the production of the antiserum might be inaccessible in the native protein due to specific folding of the receptor, the interaction with auxiliary factors, or the formation of oligomers as shown for bitter receptors [12], [17], [31], or any other reason. The visualization of such less accessible epitopes may require a careful and individual adjustment of the experimental conditions such as variations in buffer constituents, pH-values, detergent use, fixation conditions or the utilization of antigen retrieval procedures frequently performed in immunogenic detection experiments. Thus, it would be premature to conclude that the antisera that failed to detect the corresponding receptors in our initial immunocytochemical screening, may not be useful in other studies. However, the successful utilization of these antibodies in future studies will require considerable efforts in the adjustment of suitable experimental conditions for each antibody.

Another reason for the observed lack of antigen recognition may however result from topological specificities of bitter taste receptors. Due to the fact that TAS2Rs belong to the family of seven transmembrane receptors, large parts of the proteins reside in form of α-helical hydrophobic domains within the lipid bi-layer of cellular membranes and are therefore less useful for the production of antisera. Because the intracellular loops of the TAS2Rs are less diverse among each other than the extracellular domains [32] and likely interact with cellular proteins [33], [34], the extracellular domains appear favorable for deriving potential immunogenic peptides. As the average amino termini of TAS2Rs are short, the extracellular loops should be the most favorable regions for the detection of the majority of human bitter taste receptors. Indeed, although the manufacturers of the chosen anti-TAS2R antibodies were rather reluctant with informations regarding the exact location of the chosen epitopes, all antibodies were raised against extracellular domains (approximate location of epitopes: anti-TAS2R3, aa 120–220, extracellular; anti-TAS2R4, aa 100–200, extracellular; TAS2R38, aa 200–300, extracellular; TAS2R43, aa 140–190, extracellular; TAS2R46, aa 50–100, extracellular; personal communication). Thus, of the five used antisera, three, anti-TAS2R3, -R4, and -R43 targeted the 2^nd^ extracellular loops of the corresponding receptors, whereas the anti-TAS2R38 and anti-TAS2R46 antisera should recognize the 3^rd^ and 1^st^ extracellular loops, respectively. As we have shown recently, all 25 functional human TAS2Rs possess a highly conserved consensus sequence for Asn-linked glycosylation required for function in all example receptors tested [3], it appears likely that all TAS2Rs are posttranslationally modified within this area frequently chosen as immunogenic peptides (cf. anti-TAS2R3, -R4, -R43 antisera in this report). Thus, the presence of a large, bulky oligosaccharide side-chain not present in synthetic peptides used for the generation of the corresponding antisera may interfere with the efficient recognition of TAS2Rs.

For the TAS2R38 antiserum, which was raised against a peptide derived from the 3^rd^ extracellular loop, we demonstrated that it recognized the corresponding receptor in the native state when overexpressed (Fig. 1 and Fig. 3) and after denaturation on Western blots (Fig. 4A). This raised the interesting possibility that this antiserum may also interfere with the activation of TAS2R38 in functional assays and thus, may allow its utilization as pharmacological tool. Although our functional calcium imaging experiments indeed demonstrated a statistically significant reduction of TAS2R38-mediated signals by the highest antibody concentration used in our experiment (Fig. 4B), the antiserum also reduced, by trend, signals obtained with a different receptor (TAS2R10). Therefore, unlike the anti-TAS2R10 antiserum that was used to suppress strychnine-stimulated calcium signals [20], the TAS2R38 antiserum is rather inactive in that respect. Whether this lack of interference in functional assays might be due to differences in the activation mechanisms of TAS2R10 and TAS2R38, different epitopes bound by the antibodies, or different sensitivities of endogenously and heterologously expressed receptors for exogenous interference, remains to be determined.

A major problem in the earlier years of bitter taste receptor research was the difficulty to functionally express the receptors in heterologous cells [5]. Like in the case of odorant receptors [35], [36], a lack in cell surface localization of the heterologously expressed TAS2R molecules in mammalian cell lines was observed and believed to be, at least in part, responsible for difficulties in functional expression systems. Indeed, this problem was solved by the modification of receptors by the addition of N-termini of other GPCRs [5], [7]. Later, it turned out that not all bitter taste receptors required such modifications for function in mammalian cell lines [17] and that other factors, or the absence thereof, might contribute to this phenomenon [17], [31]. Interestingly, the subcellular distribution of TAS2R38 in human circumvallate papillae (Fig. 5) clearly indicated a significant intracellular localization of this receptor. Hence, what might be considered to indicate trafficking problems or overexpression effects *in vitro*, actually resembles, at least on a macroscopic scale, the receptor distribution as seen *in vivo*. Of course, even if only a fraction of the total amount of receptors present at the cell surface is required for normal function *in vivo*, some heterologously expressed native TAS2Rs may have fewer than necessary. On the other hand, it appears likely that a number of other factors contribute to the functionality of TAS2Rs even if the presence at the cell surface is not in all cases the limiting factor such as the direction to cellular subdomains [31], chaperone assisted folding [3], etc.

Our immunocytochemical experiment using the TAS2R38-antiserum directed against an extracellular epitope of the receptor and the anti-HSV antiserum recognizing an intracellularly localized epitope in parallel may provide a hint to the dynamics of bitter receptor biosynthesis and routing. While we observed a complete overlap of the two detected epitopes on a cellular level, on a subcellular level differences in the distribution of the signals became apparent. Bitter taste receptors are subjected to glycosylation [3], they oligomerize [12], interact with the endoplasmic reticulum-resident chaperone calnexin [3], associate with G proteins [37]–[39] and auxiliary factors [17], [31]. All of these processes, as well as additional posttranslational modifications, which are likely to occur in addition, could selectively affect the accessibility and hence detection efficiency of extra- and intracellular epitopes.

In the future, the availability of specific antibodies against bitter taste receptors will hopefully allow filling many gaps in our current knowledge about TAS2R expression (e.g. in gastrointestinal tissues), function, cell biology, and biochemistry.

## Materials and Methods

### Ethics Statement

The collection of biopsy material of patients was approved by the local ethics committee (Charite, Berlin, appl. #240/2002) and written informed consent was obtained from all participants. Samples were analyzed anonymously.

### Immunocytochemistry

Immunofluorescent detection of bitter taste receptor transfected cells was mainly performed as described previously [10]. Briefly, HEK 293T cells stably expressing the G protein-chimera Gα16gust44 were seeded onto poly-D-lysine coated glass cover slips. Next, cells were transiently transfected using either Lipofectamine 2000 (Invitrogen) or FuGene HD (Roche) according to the manufacturer’s protocols with cDNA constructs coding for the human bitter taste receptors TAS2R3, -R4, -R7, -R14, -R16, -R38, R41, -R43, -R46 or the mouse bitter taste receptor Tas2r138. The coding regions of the receptors were fused N-terminally to a sst3-epitope and C-terminally to either a FLAG-tag (TAS2R46) or a HSV-tag (all other receptor constructs). Empty pcDNA5/FRT vector (Invitrogen) served as negative control. After 24 h at 37°C, 5% CO_2_, cells were washed with PBS, cooled on ice for 30 min and incubated with biotin-conjugated concanavalin A (5 µg/mL, Sigma) for 1 h. Next, cells were washed with PBS, fixed by methanol:acetone (1∶1, v/v) treatment, washed again with PBS and incubated in PBS containing 5% normal horse serum before primary antibodies were added at concentrations summarized in table 1 and left on the cells overnight at +4°C. Cells were then washed with PBS, blocked for one hour in PBS containing 5% normal horse serum and incubated with the appropriate secondary antibodies (Table 1) for 1 h at room temperature. Finally, cells were washed, embedded with Fluorescent Mounting Medium (Dako) and analyzed by confocal laser-scanning microscopy (Leica TCS-SP2).

**Table 1:** Primary and secondary antibodies used in this study.

Primary antibodies				
Specificity	Supplier	Product code	Species/type	Dilution(s)
Human TAS2R3	Thermo Scientific	OSR00152W	Rabbit/polyclonal	**1∶300** (IC), 1∶1000
Human TAS2R4	Thermo Scientific	OSR00153W	Rabbit/polyclonal	**1∶300** (IC), 1∶800
Human TAS2R38	Abcam	Ab65509	Rabbit/polyclonal	1∶500, 1∶800, 1∶1500, 1∶3000, **1∶3500** (IC), **1∶4000** (IHC, WB)
Human TAS2R43	Santa Cruz Biotechnology	Sc-34850	Goat/polyclonal	1∶50, **1∶250** (IC)
Human TAS2R46	Santa Cruz Biotechnology	Sc-34732	Goat/polyclonal	1∶50, 1∶100, **1∶250** (IC), 1∶500
HSV-epitope	Novagen	69171	Mouse/monoclonal	**1∶15000** (IC)
FLAG-epitope	Sigma-Aldrich	F7425	Rabbit/polyclonal	**1∶2000** (WB, IC)
PLC β2 (Q-15)	Santa Cruz Biotechnology	Sc-206	Rabbit/polyclonal	**1∶100** (IHC)
Secondary antibodies				
Alexa Fluor488 goat anti-mouse IgG (H+L)	Molecular Probes	A-11029	Goat/polyclonal	1∶2000
Alexa Fluor488 goat anti-rabbit IgG (H+L)	Molecular Probes	A-11034	Goat/polyclonal	1∶1000
Alexa Fluor488 donkey anti-goat IgG (H+L)	Molecular Probes	A-11055	Donkey/polyclonal	1∶2000
Alexa Fluor546 goat anti-mouse IgG (H+L)	Molecular Probes	A-11003	Goat/polyclonal	1∶2000
Fluorescein goat anti-rabbit IgG (H+L)	Vector Laboratories	FI-1000	Goat/polyclonal	1∶2000

The antibodies are listed together with their specificities, suppliers, product codes, and the species and type of antibody. The antibody dilutions that were tried are shown in the right column. Dilutions printed in bold were used for experiments shown in the manuscript together with the corresponding procedure (IC  =  immunocytochemistry, IHC  =  fluorescent immunohistochemistry, WB  =  Western blotting).

### Immunoprecipitation and Western Blotting

Preparation of cell extracts, immunoprecipitation and Western blotting were mainly done as described previously [17] except that plasma protein extracts were used for immunoprecipitation. Briefly, HEK 293T-Gα16gust44 were transiently transfected with TAS2R16 and TAS2R38 constructs in which the coding regions of the bitter taste receptors were N-terminally extended by an sst3-epitope and C-terminally by a FLAG-tag using Lipofectamine 2000 (Invitrogen). After 24 h cells were harvested and subsequently homogenized on ice in hypotonic buffer (50 mM Tris-HCl, 10 mM NaCl, 2 mM EDTA, 1 mM PMSF, 2 µg/ml Leupeptin, 2 µg/ml Pepstatin A; pH 8.0). Following a 5 min centrifugation (≤50g, +4°C), the supernatants were transferred into fresh tubes and subjected to high-speed centrifugation (2 h, 50000 g, +4°C). The supernatants were removed and the pellets were washed with hypotonic buffer and centrifugated again (1 h, 50000g, +4°C). Next, the pellets were resuspended in extraction buffer (1x TBS, 1 mM PMSF, 2 µg/ml Leupeptin, 2 µg/ml Pepstatin A, 1% Triton X-100, 0.5% sodium deoxycholate; pH 7.5) and kept on ice for 30 min. After another centrifugation (30 min, 16000g, +4°C), protein contents of the supernatants containing the extracted membrane proteins were determined by a modified Bradford-assay (BioRad) and 255 µg of extracted protein each was subjected to immunoprecipitation using anti-FLAG agarose (overnight, +4°C, on a rotary wheel). Agarose-bound proteins were harvested by centrifugation, washed, mixed with standard SDS-PAGE-loading buffer without reducing agents and eluted from the agarose-beads by 30 min incubation at 56°C. Prior to SDS-PAGE, dithiotreitol (DTT) was added to a final concentration of 200 mM and samples were heated for 5 min at 95°C. SDS-PAGE and Western blotting were performed according to standard procedures. For the detection of FLAG-tagged receptor proteins rabbit anti-FLAG antibody (1∶2000, Sigma) in combination with an alkaline phosphatase conjugated sheep anti-rabbit secondary antiserum (1∶5000, Sigma) was used. Detection of TAS2R38 was achieved using a rabbit anti-TAS2R38 antiserum (1∶4000, Abcam) and a sheep anti-rabbit alkaline phosphatase antibody (1∶5000, Sigma). Blotting membranes were stained by standard colorimetry.

### Immunohistochemistry

Ten µm cross-sections of human circumvallate papillae were cut with a cryostat, thaw-mounted onto positively charged glass slides and stored at −80°C. Next, slides were fixed in freshly prepared 4% paraformaldehyde in 1x phosphate-buffered saline (PBS; 20 mM NaH_2_PO_4_, 80 mM Na_2_HPO_4_, 65 mM NaCl, pH 7.4), pH7.2, for 5 min at room temperature. Following three rinses in 1x PBS for 5 min at room temperature each, slides were blocked in 1x PBS, 10% normal horse serum, 0.5% Triton X-100 for 1 h at room temperature. The anti-TAS2R38 antiserum was applied to the sections at a dilution of 1∶4000 in a buffer consisting of 1x PBS, 5% normal horse serum, 0.2% Triton X-100 and allowed to react overnight at +4°C. For negative control the primary antibody was pre-incubated with a 5-fold excess (v/v) of the immunogenic peptide and used in the same buffer on adjacent sections. On the next day, slides were washed three times in 1x PBS (5 min each, room temperature), incubated with secondary antibody (1∶2000, anti-rabbit Fluorescein) in 1x PBS, 5% normal horse serum, 0.2% Triton X-100 for 1 h at room temperature, and rinsed 3-times in PBS (5 min each, room temperature) before an antiserum specific for the type II taste cell marker PLCβ2 was applied. In order to circumvent problems arising from the fact that both primary antisera were generated in the same species (rabbit), we labeled the PLCβ2 antibody directly using the Zenon antibody labeling kit according to the manufacturers’ protocol (Zenon Alexa Fluor 647 Rabbit IgG Labeling Kit, Invitrogen). PLCβ2-Zenon-complex obtained from the labeling of 1 µg PLCβ2-antibody was added in 1x PBS, 5% normal horse serum, 0.2% Triton X-100 and left on the sections for 60 min at room temperature. For negative control an identical labeling reaction was pre-incubated with 3 µg of the antigenic blocking peptide and applied onto adjacent sections. Next, the slides were washed three times with 1x PBS (5 min each, room temperature). After postfixation (4% paraformaldehyde in 1x PBS, pH 7.2, 15 min, room temperature), sections were rinsed with ddH_2_O, embedded in Fluorescent Mounting Medium (Dako) and analyzed by confocal laser-scanning microscopy (Leica TCS-SP2). For a slightly deviating protocol used for the immunohistochemical analyses of mouse vallate papillae sections see Materials and Methods S1.

### Functional Calcium Imaging Experiments

Functional analyses of bitter taste receptors in the presence of the specific TAS2R38 antibody was essentially performed as before (e.g. [2], [10], [17]). Briefly, HEK 293T-Gα16gust44 cells were transiently transfected with TAS2R38 or TAS2R10 (negative control) using Lipofectamine 2000. After 24 h, the cells were loaded with Fluo4-AM and washed twice with buffer C1. Instead of rinsing the cells a third time with C1 buffer, C1 buffer containing different dilutions of the TAS2R38-antiserum was applied and left on the cells for 30 min prior to the application of agonists. Next, the cells were placed into a fluorometric imaging plate reader and the baseline fluorescence was measured. The changes in fluorescence upon agonist stimulation at fixed concentrations of 30 µM phenylthiocarbamide (PTC) and 100 µM denatonium benzoate for the TAS2R38 and TAS2R10, respectively, were monitored and corrected for basal fluorescence. The relative changes in fluorescence (ΔF/F) obtained for receptor transfected cells not treated with the TAS2R38 antibody were set as 100% and used for comparisons.

## Supporting Information

Materials and Methods S1 Immunohistochemical analyses of mouse vallate papillae sections.
(DOC)

Figure S1 **Screening for cross-reactivity of the TAS2R38 antiserum with TAS2Rs (part 1).** HEK 293T-Gα16gust44 cells were transiently transfected with cDNA coding for the human bitter taste receptors TAS2R1, -R5, -R20, -R30, -R31, -R39, -R40, and -R42. To visualize the receptor proteins (receptor, green) either antibodies against the C-terminal HSV-tag (left panels, epitope tag) or the TAS2R38-specific antiserum (right panels, TAS2R38) were used. The cell surfaces were labeled with biotin-conjugated concanavalin A (cell surface, red). Overlay pictures of the merged green and red channels are shown (overlay). Scale bar, 50 µm.
(TIF)

Figure S2 **Screening for cross-reactivity of the TAS2R38 antiserum with TAS2Rs (part 2).** HEK 293T-Gα16gust44 cells were transiently transfected with cDNA coding for the human bitter taste receptors TAS2R50 and -R60. To visualize the receptor proteins (receptor, green) either antibodies against the C-terminal HSV-tag (left panels, epitope tag) or the TAS2R38-specific antiserum (right panels, TAS2R38) were used. The cell surfaces were labeled with biotin-conjugated concanavalin A (cell surface, red). Overlay pictures of the merged green and red channels are shown (overlay). Scale bar, 50 µm.
(TIF)

Figure S3 **Immunohistochemistry of mouse vallate papillae sections.** In order to identify whether the human anti-TAS2R38 antiserum, which does not recognize the orthologous mouse receptor Tas2r138, reacts with other antigens present in mammalian gustatory tissue, we performed immunohistochemical experiments using anti-TAS2R38 in combination with different secondary antisera. A, section of mouse vallate papillae (VP) stained with TAS2R38 antiserum and goat anti-rabbit Alexa Fluor488 (1∶1000, Molecular probes). Note green fluorescent signals outlining the trench of the VP as well as in the pore region of taste buds. Some signals detected in the taste pores extend into the taste bud (inset). B, negative control for A obtained by pre-absorption of TAS2R38 antiserum with antigenic peptide. Note that signals seen in A persist indicating unspecific staining including the signals found in and extending from pore region (inset, scale bar = 10 µm). C, same as A except that the TAS2R38 antiserum was used in combination with sheep anti-rabbit Cy3 (1∶2000, Sigma). Note that in contrast to A only some taste pores are faintly stained and signals do not extend into the taste bud. D, negative control for C obtained by pre-absorption of TAS2R38 antiserum with antigenic peptide. Note that immunoreactivity in taste pores is not diminished by peptide blocking. G-I, section of mouse VP co-stained with TAS2R38 antiserum in combination with goat anti-rabbit Fluorescein secondary antibody (1∶2000, Sigma) and Alexa Fluor647 labeled PLC β2 antiserum. Note the absence of intragemmal TAS2R38-immunoreactivity (G, green), while PLC β2-specific staining is evident in taste bud cells (H, red). The overlay of green and red channels shows that only the pore regions are double-stained by the used antisera (I). J-L, negative controls for G-I obtained by pre-absorption of TAS2R38- and PLC β2-antisera with the corresponding antigenic peptides. Scale bars, 50 µm.
(TIF)
