# Supplementary material for: Immunohistochemical Detection of TAS2R38 Protein in Human Taste Cells
Source: PLoS One. 2012 Jul 6;7(7):e40304. doi: 10.1371/journal.pone.0040304 (PMC3391245; doi:10.1371/journal.pone.0040304)
Supplement: Materials and Methods S1 — Immunohistochemical analyses of mouse vallate papillae sections. (DOC) [file pone.0040304.s001.doc]

**Materials and Methods S1**

Immunohistochemistry of mouse vallate papillae sections. The immunohistochemical analyses of mouse vallate papillae sections were mainly done as described for human gustatory tissue in the Material and Methods section of the main manuscript. Vallate papillae of C57BL/6 mice were cut with a cryostat to 14 µm thickness. The following antisera were used in combination with the polyclonal rabbit TAS2R38 antiserum (Abcam, Ab65509, 1:4000 dilution): 1. goat anti-rabbit Alexa Fluor488 (Molecular probes, A-11034, 1:1000 dilution); 2. sheep anti-rabbit F(ab)_2_ Cy3 (Sigma-Aldrich, C2306, 1:2000 dilution); 3. goat anti-rabbit Fluorescein (Vector Laboratories, FI-1000, 1:2000 dilution); 4. rabbit anti-PLC β2 (Q-15) (Santa Cruz Biotechnology, sc-206, 1:100 dilution, directly labeled with Zenon Alexa Fluor 647 Rabbit IgG Labeling Kit, Invitrogen (see main manuscript)). For the negative control reactions primary antibodies were pre-absorbed with the corresponding antigenic peptides (anti-TAS2R38 antiserum was incubated with a 5-fold access of antigenic peptide, 1 µg Zenon-labeled anti-PLC β2 antiserum with 3 µg blocking peptide).
