# Supplementary figures and images for: Immunohistochemical Detection of TAS2R38 Protein in Human Taste Cells
Source: PLoS One. 2012 Jul 6;7(7):e40304. doi: 10.1371/journal.pone.0040304 (PMC3391245; doi:10.1371/journal.pone.0040304)

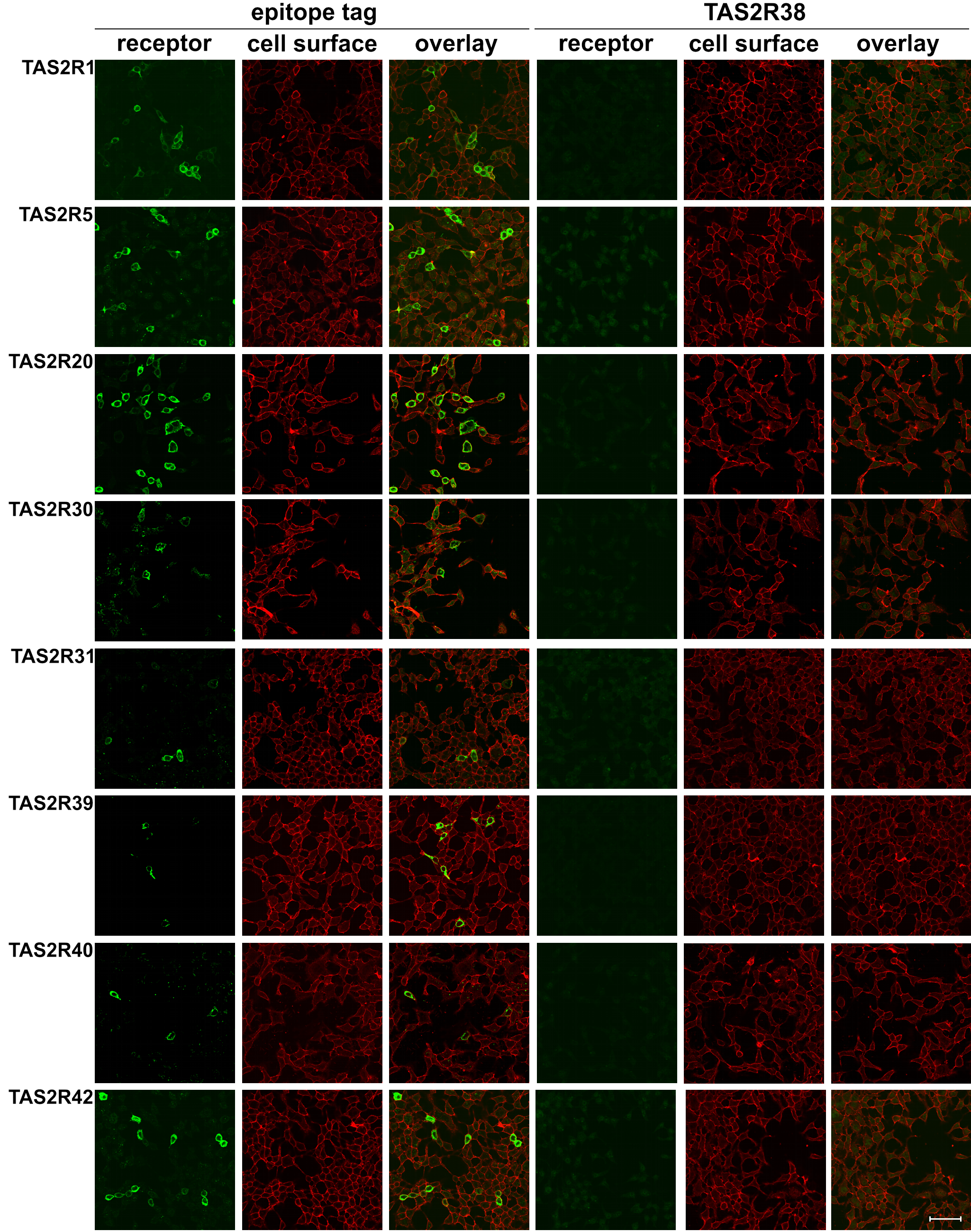

Supplement: Figure S1 — Screening for cross-reactivity of the TAS2R38 antiserum with TAS2Rs (part 1). HEK 293T-Gα16gust44 cells were transiently transfected with cDNA coding for the human bitter taste receptors TAS2R1, -R5, -R20, -R30, -R31, -R39, -R40, and -R42. To visualize the receptor proteins (receptor, green) either antibodies against the C-terminal HSV-tag (left panels, epitope tag) or the TAS2R38-specific antiserum (right panels, TAS2R38) were used. The cell surfaces were labeled with biotin-conjugated concanavalin A (cell surface, red). Overlay pictures of the merged green and red channels are shown (overlay). Scale bar, 50 µm. (TIF) [file pone.0040304.s002.tif]

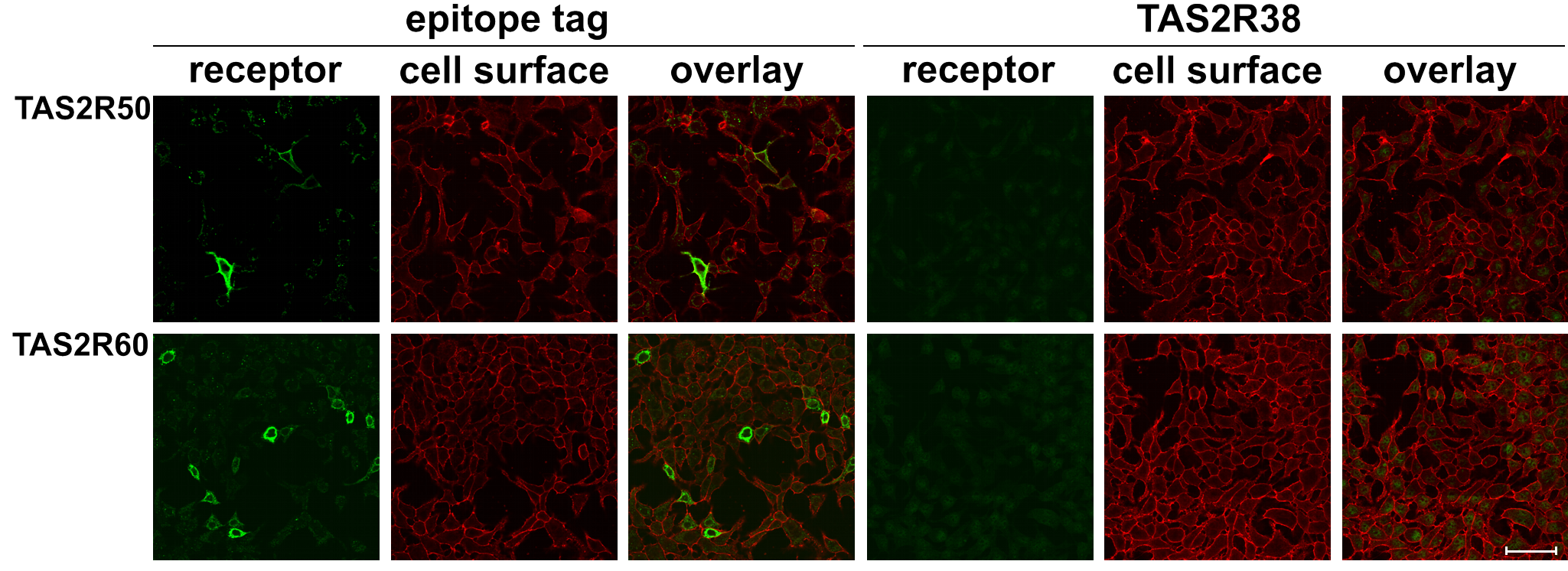

Supplement: Figure S2 — Screening for cross-reactivity of the TAS2R38 antiserum with TAS2Rs (part 2). HEK 293T-Gα16gust44 cells were transiently transfected with cDNA coding for the human bitter taste receptors TAS2R50 and -R60. To visualize the receptor proteins (receptor, green) either antibodies against the C-terminal HSV-tag (left panels, epitope tag) or the TAS2R38-specific antiserum (right panels, TAS2R38) were used. The cell surfaces were labeled with biotin-conjugated concanavalin A (cell surface, red). Overlay pictures of the merged green and red channels are shown (overlay). Scale bar, 50 µm. (TIF) [file pone.0040304.s003.tif]

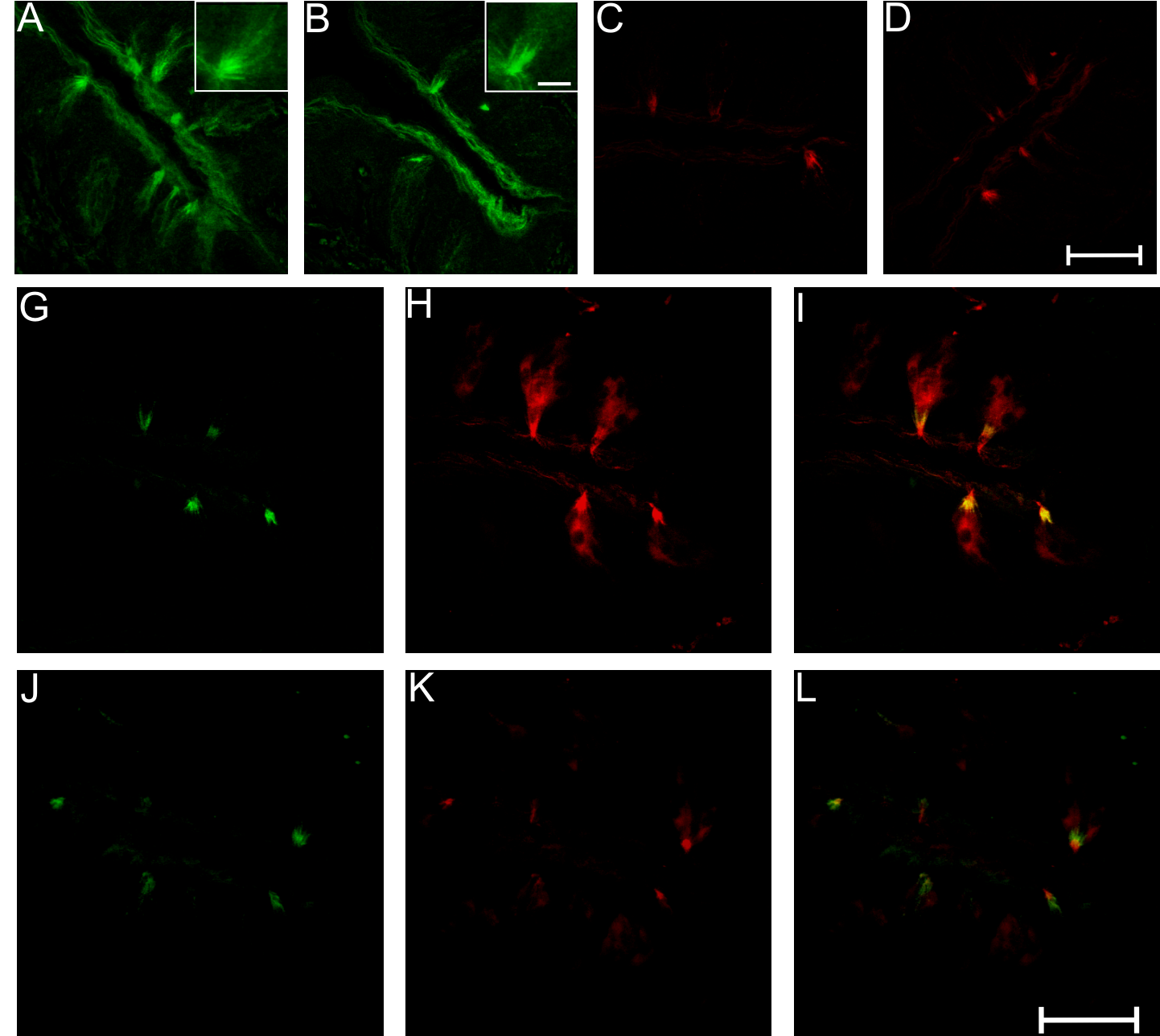

Supplement: Figure S3 — Immunohistochemistry of mouse vallate papillae sections. In order to identify whether the human anti-TAS2R38 antiserum, which does not recognize the orthologous mouse receptor Tas2r138, reacts with other antigens present in mammalian gustatory tissue, we performed immunohistochemical experiments using anti-TAS2R38 in combination with different secondary antisera. A, section of mouse vallate papillae (VP) stained with TAS2R38 antiserum and goat anti-rabbit Alexa Fluor488 (1∶1000, Molecular probes). Note green fluorescent signals outlining the trench of the VP as well as in the pore region of taste buds. Some signals detected in the taste pores extend into the taste bud (inset). B, negative control for A obtained by pre-absorption of TAS2R38 antiserum with antigenic peptide. Note that signals seen in A persist indicating unspecific staining including the signals found in and extending from pore region (inset, scale bar = 10 µm). C, same as A except that the TAS2R38 antiserum was used in combination with sheep anti-rabbit Cy3 (1∶2000, Sigma). Note that in contrast to A only some taste pores are faintly stained and signals do not extend into the taste bud. D, negative control for C obtained by pre-absorption of TAS2R38 antiserum with antigenic peptide. Note that immunoreactivity in taste pores is not diminished by peptide blocking. G-I, section of mouse VP co-stained with TAS2R38 antiserum in combination with goat anti-rabbit Fluorescein secondary antibody (1∶2000, Sigma) and Alexa Fluor647 labeled PLC β2 antiserum. Note the absence of intragemmal TAS2R38-immunoreactivity (G, green), while PLC β2-specific staining is evident in taste bud cells (H, red). The overlay of green and red channels shows that only the pore regions are double-stained by the used antisera (I). J-L, negative controls for G-I obtained by pre-absorption of TAS2R38- and PLC β2-antisera with the corresponding antigenic peptides. Scale bars, 50 µm. (TIF) [file pone.0040304.s004.tif]
